# Patient engagement in protocol development for rare disease clinical trials: a framework

**DOI:** 10.1186/s40900-026-00985-3

**Published:** 2026-10-09

**Authors:** Janella Beltran, Sarah Bolton, Pesh Patel, Rohita Sharma

**Affiliations:** 1 Global Patient Experience – Medical Affairs, Alexion, AstraZeneca Rare Disease, 1004 Middlegate Rd, Mississauga, ON L4Y 1M4 Canada; 2 San Luis Obispo, CA USA; 3 Lafayette, LA USA

**Keywords:** Clinical trial, Patient perspective, Rare disease, Trial design

## Abstract

**Background:**

Meaningful patient engagement requires understanding lived disease and healthcare experiences. This patient-centric approach is especially valuable when applied to the design and execution of clinical trials, as participation barriers may hinder trial recruitment, retention, and detection of meaningful outcomes. Patient engagement is particularly important in the study of treatments for rare diseases, where populations are small and geographically dispersed and there are few sites with relevant clinical expertise. Additionally, protocol amendments to address hurdles are expensive and can cause delays in trial activities, which can affect downstream regulatory approvals and access to treatments. Alexion, AstraZeneca Rare Disease, worked with patients and their stakeholders to identify points of additional trial burden and enable patient-driven changes before clinical trial protocol finalization.

**Methods:**

Patients, parents of pediatric patients, and patient advocacy group representatives with diverse experiences participated in virtual, study-specific perspective exchange sessions on 4 protocols (adults with generalized myasthenia gravis, pediatric patients with generalized myasthenia gravis, adults with antibody-mediated rejection after kidney transplantation, and adults with transthyretin amyloid cardiomyopathy). Participants discussed their disease journey, the protocol, and perceived barriers to clinical trial awareness, enrollment, participation, and follow-up through semistructured interviews.

**Results:**

Participants raised points about the eligibility criteria, study drug self-administration, visit frequency, participant mental health, and the relevance of specific outcome measures. In response, the study sponsor proactively changed the clinical trial protocols when feasible. Changes included expanding the eligibility criteria, developing an instructional video on self-administration of the study drug, and reducing the frequency of clinic visits. In addition, the sponsor created appreciation cards and trained trial sites on a standardized approach to help participants understand the goals of specific outcome measures.

**Conclusions:**

These insights led to demonstrable, advocate-recommended changes in rare disease clinical trial protocols before study delivery, underscoring how early patient-partnered drug development can be meaningfully applied to research in rare diseases.

**Trial registration:**

NCT05556096, NCT06607627, NCT06744647, and NCT06183931.

**Clinical trial number:**

Not applicable.

**Supplementary Information:**

The online version contains supplementary material available at https://doi.org/10.1186/s40900-026-00985-3.

## Abstract (plain language summary)

Running clinical trials in rare diseases can be difficult for a number of reasons: (1) recruiting patients can be a challenge because there are so few people with any one rare disease; (2) there isn’t much information about which study goals will make a difference for patients; and (3) patients often drop out of trials, which take a lot of time and effort. When changes are needed to address these challenges, researchers have to change the design of the trial after it has started. This is time consuming, expensive, and can delay treatment approval and patient access. The goal of this initiative was to show that if patients were involved in trial design prior to study initiation, the study organizer would be able to address potential issues before the trial started. Patients and their caregivers provided input into the design of 4 trials during virtual meetings with researchers. Participants talked about what parts of the trial would make it hard for people to participate and whether they thought the trial was answering the right questions. For example, participants raised concerns about how often they would have to go for trial visits. The researchers then made changes to the trial protocols based on the feedback gathered. These patient-guided changes improved the trial design before it started. By working with patients, researchers were able to make the trials more patient friendly. The initiative found that including patient perspective was truly beneficial in the design of clinical trials.

**Supplementary Information:**

The online version contains supplementary material available at https://doi.org/10.1186/s40900-026-00985-3.

## Background

Government agencies, advocacy organizations, and key opinion leaders emphasize patient engagement during drug development. For instance, the 21st Century Cures Act (enforced by the US Food and Drug Administration) provides guidance on patient-focused drug development to ensure that patient perspectives are considered during trial design, regulatory decisions, and risk-benefit assessments. Similarly, the European Medicines Agency published a framework for engagement with patients, consumers, and their organizations [1]. While the importance of patient involvement in research and drug development is globally recognized, the actual practice of consistent engagement remains a challenge and requires a cultural shift in the industry.

Meaningful engagement with patients during drug development involves gaining a deep, personalized understanding of their lived disease and healthcare experiences [2] and willingness to cocreate strategies and solutions [3]. This patient-centric approach is especially valuable when applied to the design and execution of clinical trials because it delivers greater value for patients by creating efficiency in stages as early as recruitment through to the consideration of meaningful outcomes and access to treatments [4]. Patients have expressed that barriers affecting participation in clinical trials include the perceived burden of participation (e.g., hospital visits, time spent completing questionnaires, and out-of-pocket costs) and the feeling of being a research “subject” [4]. Failing to understand these barriers may result in poor recruitment and retention (i.e., participant dropout), which may underpower a clinical trial and negatively impact the ability to detect statistically significant or clinically meaningful outcomes [5].

The burden of patient participation is particularly important in the study of treatments for rare diseases, where patient populations are small and frequently geographically dispersed, complicating patient identification, recruitment, and retention [6, 7]. There are also few clinical sites with relevant expertise for rare diseases, adding to concerns about travel for patients with impairments and their families [8]. In addition, the ethics of administering a placebo to patients with potentially shortened lifespans and/or rapidly progressing diseases must be considered [9], and high, interindividual variability in clinical course and a lack of validated measures for disease activity may affect the design of statistically and clinically meaningful trials [9]. Despite agreement that patient involvement in research is important, there is a need for identifying meaningful partnership methods [9], especially for rare diseases.

Some of these challenges may be alleviated by clinical trial protocol amendments. The most common amendment is the addition of clinical sites, and the most common reason for amendments is to achieve recruitment targets [10]. However, clinical trial protocol amendments are expensive and can cause delays in trial activities, which can affect downstream regulatory approvals and access to treatments [11]. A systematic assessment of clinical trial protocol elements (e.g., assessment schedules) has been proposed to understand the burden imposed on trial participants and to identify opportunities to optimize trial design and execution [12]. The resulting patient friction coefficient is a customizable index that determines the impact of protocol specifications on perceived patient burden by considering cost, time, and degree of daily routine disruption [13]. The patient friction coefficient was developed and tested using sample sizes larger than those available for clinical trials in rare disease [13]. As a result, the patient friction coefficient is not suitable for the evaluation of participation in rare disease clinical studies given smaller patient populations and limited availability of real-world data on clinical practices to quantify or establish a numeric score related to trial participation [13]. Due to this limitation, Alexion, AstraZeneca Rare Disease, has adapted this approach to develop patient perspective exchange sessions (also referred to as “patient perspective exchanges”), which allow direct engagement with patients, their caregivers, and patient advocacy group representatives. The purpose of this initiative was to demonstrate that patient perspective exchanges can be applied to clinical trial design and study implementation. Ultimately, this work seeks to encourage other sponsors to adopt a patient-focused approach to clinical development by demonstrating that patients can be engaged before protocol finalization to inform trial design and proactively identify where a protocol amendment may need to occur, with the goal of improving trial efficiency.

## Methods

### Protocol concept sheets selected for exchanges

Alexion, AstraZeneca Rare Disease includes patient and caregiver insights in protocol development. As of October 17, 2024, patient and caregiver insights prior to clinical trial protocol finalization have been collected for 11 clinical trials. In this report, we describe the methodology and results pertaining to 4 of these protocols. These protocols were selected based on the robustness of the insights that were gathered from the patient perspective exchanges. Only trials nearing completion and regulatory approval were included due to confidentiality associated with in progress trials. These trials are representative of sessions held across all phase 3 trials led by the sponsor.

The first protocol describes a phase 3 trial of ALXN1720 (gefurulimab) in the treatment of adults with generalized myasthenia gravis (ClinicalTrials.gov clinical trial number: NCT05556096; first posted 2022-09-27) [13]. The second protocol describes a phase 3 trial of gefurulimab in pediatric patients (aged 6 to <18 years) with generalized myasthenia gravis who express acetylcholine receptor antibodies (ClinicalTrials.gov clinical trial number: NCT06607627; first posted 2024-09-23) [14]. The third protocol describes a phase 2 trial of ALXN2030 in adults with active or chronic active antibody-mediated rejection (AMR) after kidney transplantation (ClinicalTrials.gov clinical trial number: NCT06744647 [CONCORD]; first posted 2024-12-20) [15]. The fourth protocol describes a phase 3 trial of ALXN2220 in adults with transthyretin amyloid cardiomyopathy (ClinicalTrials.gov clinical trial number: NCT06183931; first posted 2023-12-28) [16]. Additional details about these clinical trials are provided in Table S1.

### Ethics

These sessions were conducted as a patient engagement activity intended to inform protocol development and trial design, rather than as a formal qualitative research study. As such, while the trials received ethical approvals, formal ethics committee review was not sought for the patient perspective exchanges. All activities were conducted in accordance with applicable company policies for patient engagement, and sponsor staff involved completed required ethics and compliance training. Participant confidentiality was maintained by documenting feedback without attribution to individual participants, and names were not linked to the feedback provided. Participants were compensated for their time and expertise. Compensation for participation was determined based on fair market value. Participants were informed of the purpose of the sessions, the voluntary nature of participation, and how their feedback would be documented and used, including measures taken to ensure confidentiality.

### Selection and recruitment of participants for the patient perspective exchanges

A core team comprised of the sponsor’s global clinical study lead(s), global medical lead(s), patient advocacy lead(s), and patient recruitment lead(s) was assembled for each of the selected clinical trials. The core team met to align on timing, desired profiles for the patient engagement participants (e.g., appropriate patient groups and target geographies), and other logistics prior to initiating the discussions.

Persons who self-reported having the condition of interest, being a caregiver—including parents of pediatric patients—or being patient advocacy group representatives were evaluated for inclusion in the exchange sessions. Participants were recruited primarily through patient advocacy organizations based on demonstrated lived experience with the disease relevant to the protocol. While prior clinical trial participation was not mandatory, efforts were made during the recruitment process to ensure approximately half of participants had prior trial experience. In addition, participants were only included if they agreed to participate in the workshop, were engaged with patient advocacy, and reported interest in continued involvement. Participants were invited via engagements with patient advocacy groups. Participants from multiple countries were invited to ensure representation of diverse experiences reflective of the proposed demographics of the trials (e.g., country, age, race, ethnicity); engagement was prioritized in countries with anticipated clinical trial sites. Invitation acceptance rates were not tracked.

### Patient perspective exchange sessions

Participants were provided premeeting reading material approximately 1 week before their scheduled exchange session that included information about the perspective exchange session and the list of questions and topics that would be covered. All materials were provided in simplified language suitable for a lay audience. Trial-specific patient exchanges were held virtually in 1 or 2 sessions and were moderated by the sponsor’s Patient Advocacy Leads. To help mitigate power imbalances, sessions were structured as collaborative discussions, with consideration given to the ratio of sponsor attendees to participants.

Participants were first asked to describe their disease and healthcare journey. Participants for the myasthenia gravis and transthyretin amyloid cardiomyopathy trials were asked about time to diagnosis to inform recruitment and enrollment strategies. Participants in the AMR after kidney transplantation trial were asked about timing and experience after kidney transplantation to understand potential constraints to trial participation based on the transplantation itself, to tailor patient identification and recruitment strategies, and to identify opportunities for additional care during trials. To protect personal privacy, participants were encouraged to refrain from sharing previous study participation details.

To inform the participant feedback, the sponsor group presented on the study drug being developed for the treatment of the disease. All materials were presented using simplified terminology and language suitable for a lay audience, along with explanations of terminology used. Sessions were also offered in participants’ local language to facilitate comprehension of the topics discussed. Participants had the opportunity to ask questions and clarify any information presented before providing feedback. Then, the protocol concept sheet (i.e., protocol specifications) was discussed, including the lengths of the screening period, the primary evaluation period, and the extension period; frequency of study drug administration; frequency and total number of clinic visits and remote visits; and study assessments (eligibility, demographics and clinical characteristics, efficacy, exploratory, laboratory, and safety). The medical lead for the study emphasized what changes could and could not be considered for the protocol.

After the presentation, the participants exchanged insights by following a semistructured discussion guide (Table S2). Discussion topics included clinical trial awareness to understand how participants learn about a trial; trial enrollment to understand how participants decide whether to enroll in a trial; trial participation to understand what the foreseeable challenges were with the presented study design and schedule of trial activities, or previous experiences participating in a trial, including logistics, activities, social interactions, answering questionnaires, the schedule of events, and/or self-administration of study drug; and trial follow-up to understand what the participants think about the trial experience after it ended and what their expectations are thereafter (Fig. 1). The exchanges concluded with an opportunity for the participants to propose ideas or cocreate solutions with the clinical trial sponsor employees who moderated the sessions. The sessions were not recorded to protect the confidentiality of participants and to foster comfort during discussions. Key feedback and discussions were captured by a notetaker for each session.

**Fig. 1:**
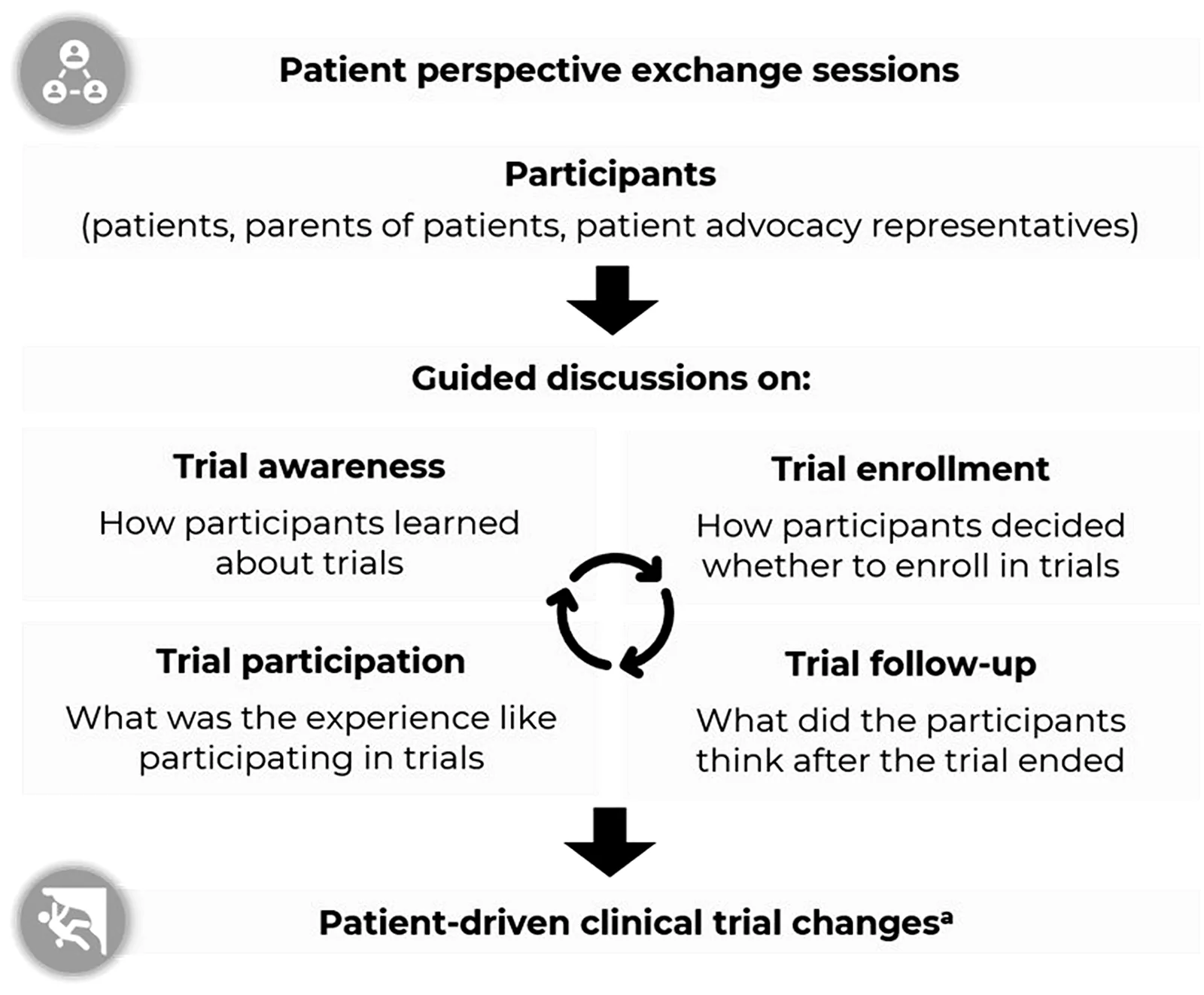
Mapping the sponsor’s approach to implementing changes in the clinical trial concept sheets based on an adapted patient friction coefficient framework. ^a^While exploring methodologies to compute clinical trial protocol complexity and patient burden, Alexion recognized that these approaches lacked patient perspective and failed to measure patient impact. Additionally, the existing frameworks were unsuitable for rare diseases due to smaller patient populations and a limited number of clinical trials in comparison to more common conditions. The proposed framework is an adaptation of the patient friction coefficient proposed by Cameron et al. [28] and incorporates the patients’ voices, measures patient impact, and is suitable for rare disease trials

### Reviewing patient perspective exchange insights and implementing changes to clinical trial protocols

Following the patient perspective exchanges, each point of feedback was discussed by the clinical development team and expert scientists across multiple internal debrief sessions to review, interpret, and address the received feedback (Fig. 2). Internal participants discussed solutions and ultimately implemented changes to the final protocol to provide support services and/or implement mitigation strategies. As the sessions were held as a structured engagement activity as part of the sponsor’s standard approach to clinical trial development and consistent with regulatory guidance, a thematic analysis was approached through the lens of feasibility. The overall timeframe for obtaining and reviewing feedback from the patient perspective exchanges occurred over approximately 6 to 8 weeks.

**Fig. 2:**
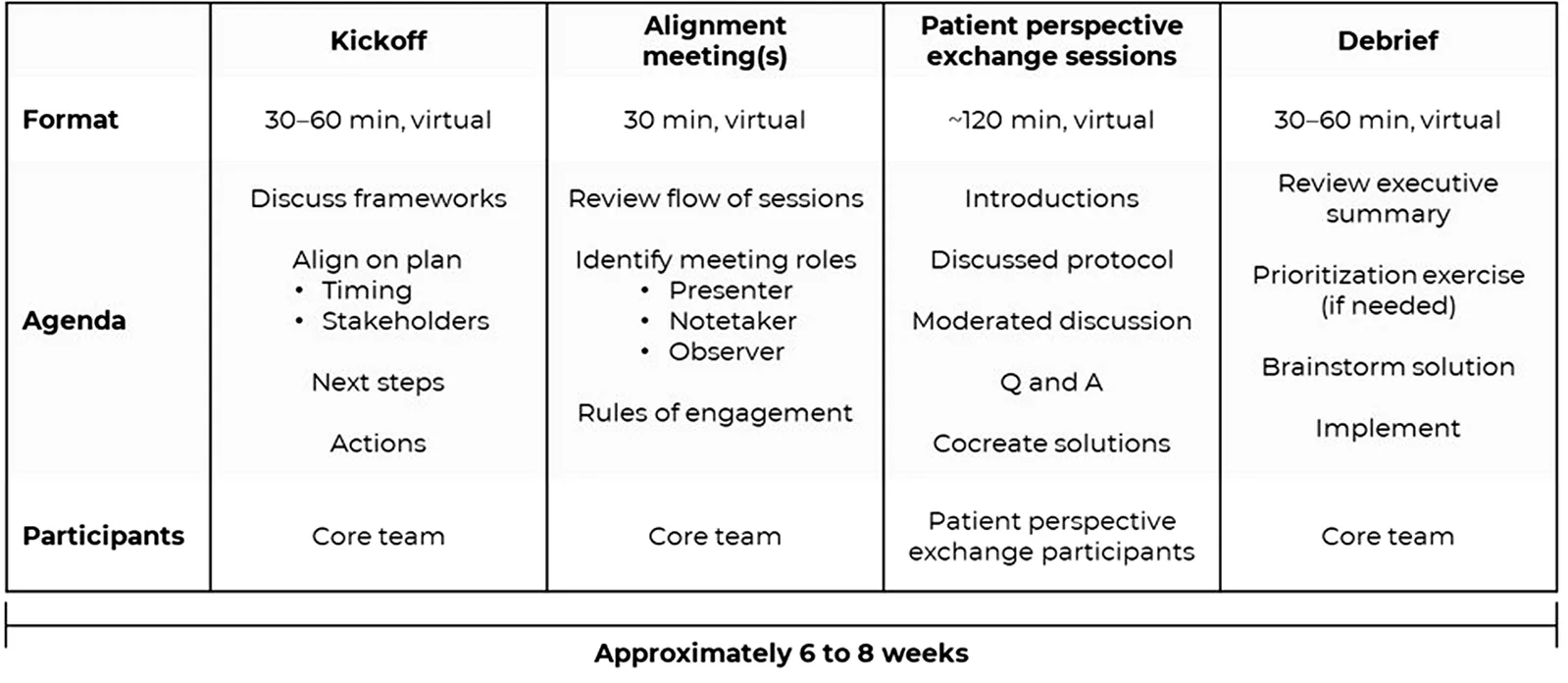
Framework for obtaining and reviewing feedback from the patient perspective exchange sessions. Desired participants for the discussions were identified during alignment meeting(s). Exchanges were held in 1 or 2 sessions. Debrief meetings could require multiple sessions. Min, minutes; Q and A, question and answer

## Results

### Patient perspective exchange participant characteristics

Four core teams (1 for each clinical trial) were assembled. A total of 22 participants were recruited for the sessions (2 parents of pediatric patients, 2 patient advocacy group representatives, 2 patient advocacy representatives who were also patients, and 16 patients with the condition of interest; Table 1). Participants were from 6 countries (Austria, France, Germany, Italy, the UK, and the US).

**Table 1:** Characteristics of the patient perspective exchange sessions and participant information

Clinical trials	Sessions per clinical trial (*n*)	Languages	Participant countries of residence	Participants (*n*)	Types of participants (*n*)
NCT05556096	1	English	US	6	• Patients: 6
NCT06607627	2	English	US	1	• Parents: 1
		Italian	Italy	2	• Parents: 1 • PAG reps: 1
NCT06744647	1	English	US	2^a^	• Patients: 2
NCT06183931	2	English	Austria, Germany, Italy, UK, US	8	• Patients: 8
		French	France	3^a^	• PAG reps: 3^b^
Totals	6	3	6	22	• Parents: 2 • PAG reps: 4^b^ • Patients: 16

“Patient” indicates an individual with the condition being investigated in the clinical trial

^a^Two additional participants were expected but could not join due to illness

^b^Two representatives were also patients

PAG rep, patient advocate group representative; UK, United Kingdom; US, United States

### Insights from the patient perspective exchanges and actions taken

Insights from the patient perspective exchanges and the subsequent sponsor actions are detailed in Table 2. Not all feedback captured resulted in a protocol change, and participants were informed during the patient perspective exchange sessions of potential limitations preventing some recommendations from being implemented.

**Table 2:** Patient perspective exchange insights and actions taken by the sponsor’s core clinical trial teams

Clinical trials	Insights	Proposed actions considered by the core clinical trial teams^a^
NCT05556096 (adults with gMG)		
Trial awareness	• Awareness comes from advocacy organizations and doctors • Encourage self-advocacy (online research, patient forums, newsletters, support groups, and social media)	• Develop an integrated patient recruitment plan encompassing both HCP awareness, patient advocacy outreach, and direct-to-patient multichannel approaches
Trial enrollment	• Concerns regarding limitations on concomitant medications, travel, and communication with trial sites • Concerns regarding previous drug exposures • Concerns over MG crisis management and response time	• Expand allowable previous drug exposures • Offer travel concierge services and reimbursement • Emphasize site staff training and support for management of crises and provide direct contact information for site staff
Trial participation	• Need for “rests” between procedures • Need for proactive and streamlined “one-stop shop” communication • Open to technological solutions (e.g., text reminders, applications, websites, and telehealth) • Strong preference to complete questionnaires at home prior to study visit • Self-administration of medications indicates independence but questions arose about self-administration	• Add “rests” between procedures in protocol • Develop instructional videos and allow for telehealth visits for study drug self-administration
Trial follow-up	• Most patients want to receive trial results from the physician or patient advocate; suggestions were a post-trial support group or calling the sponsor	• More discussion needed to address this point to ensure appropriate governance on information sharing (ongoing discussions and workstreams regarding appropriate post-trial follow-up and transparency)^b^
NCT06607627 (pediatric patients with MG)		
Trial awareness	• Awareness from clinic waiting room, social media, TV commercials for marketed products, and physicians • Participant parents noted varying degrees of knowledge of clinical trials	• Increase clinical trial awareness and foster education in pediatric setting
Trial enrollment	• Questions about placebo use, allowed concomitant therapy, vaccination requirements, and off-label medication use in children • Concerns about roundtrip family travel to the hospital; need to consider reimbursement or allowance for travel	• Concomitant medication limitations, procedures, and vaccination requirements remained. Detailed explanations were provided to ensure a clear understanding of the requirements
Trial participation	Visit schedule • General concerns about the number of visits and impact on QoL • Potential challenges for other families may be situation specific • Preferences for timing of visits varied; agreement on avoiding school disruption Self-injection • Need for additional support surrounding use of the device • Importance of training; remote access to HCPs • Importance of encouragement; injection day should be made fun	Visit schedule • Reduce clinic visit frequency and increase remote visits during open-label extension • Remove requirement for morning assessments for flexibility and convenience • Expanded support services (travel, food, hotel, and other accommodations) Self-injection • Consider optional visiting nurses to assist with initial injections at home for future studies
Trial follow-up	• Was not discussed in either session	
NCT06744647 (adults with AMR)		
Trial awareness	Kidney transplant/AMR awareness and diagnosis • Patients noted that their awareness of transplant risks is typically high level • Resort to doing their own research or ask more questions due to HCPs’ use of technical terms Trial awareness • Awareness primarily from social media, the internet, and other patients; suggested study-specific websites, social media outreach, and paid search campaigns	• Ensure AMR education is incorporated into ICFs and recruitment materials
Trial enrollment	• Patients’ advocacy for self-education and the need to receive information and resources about the disease and clinical trial opportunities o Importance of visuals in understanding information o Brochures and websites are “ideal” o Need for materials about patient mental health support • Importance of appreciation and a human component to participation in trials	• Incorporate patient feedback in the development of clinical trial recruitment materials • Ensure patient-friendly and visually engaging content in recruitment materials
Trial participation	• Transportation and distance to trial sites • No concerns with eligibility criteria, the number and length of visits, and the inclusion of vaccine requirements and questionnaires o Patients have learned to tolerate procedures o No significant concerns with periodic biopsies despite asking for alternatives • Importance of continuous patient engagement through site and sponsor communications	• Availability of home healthcare options and travel support for participants who were further from selected trial sites • Ensure that the visit guide for the in-study materials is comprehensive and clear (e.g., materials reviewed by patient advocacy lead and patient representatives) • Update protocol so that biopsies during the open-label extension period are optional and not mandatory
Trial follow-up	• Was not discussed due to time constraints	
NCT06183931 (adults with ATTR-CM)		
Trial awareness	• Awareness from nurses and HCPs • Frustrations that ClinicalTrials.gov is too technical • Frustrations due to geographic discrimination	• Develop an integrated patient recruitment, advocacy, HCP awareness plan using patient-friendly language • Travel assistance and concierge services
Trial enrollment	• Concerns include rationale for the inclusion of a placebo treatment arm, eligibility criteria, and how disease progression would be assessed	• Review clinical trial information with patients to build trust
Trial participation	• No clear understanding of the 6MWT goals • 1:1 study design was less desirable • Concerns about MRI tracer used due to potential kidney damage to patients with a heart condition • Excitement about study drug mechanism of action; clearly stating that the study drug depletes amyloid proteins would encourage patients to invest their time • Questions about IV administration • Exclusion of patients with pacemakers in the trial may not be reflective of the real-world patient population	• Vendor will train sites on the standardized approach to the 6MWT and include a script to ensure consistent understanding of goals • Change to a 2:1 (study drug to placebo) study design • Explain in the ICF and visit guide that MRI is optional (clarify the goals) • Included “amyloid depleter” in the study name • IV administration discussed with the study medical lead
Trial follow-up	• Patients ask whether the study drug worked and whether they received the drug or placebo	• Further discussion to address Clinical Trial Transparency processes (ongoing discussions and workstreams regarding appropriate post-trial follow-up and transparency)^b^

^a^Some proposed actions evolved during protocol finalization

^b^Topline results are shared in approved press releases with the patient advocacy communities as part of disease awareness outreach

6MWT, 6-minute walk test; AMR, antibody-mediated rejection; ATTR-CM, transthyretin amyloid cardiomyopathy; gMG, generalized myasthenia gravis; HCP, healthcare provider; ICF, informed consent form; IV, intravenous; MG, myasthenia gravis; MRI, magnetic resonance imaging; QoL, quality of life

#### Phase 3 study in adults with generalized myasthenia gravis (NCT05556096) [17]

Participants reported receiving information on clinical trials from advocacy organizations and doctors but also encouraged self-advocacy to search for clinical trials, as they typically consulted more than 3 healthcare providers on their path to diagnosis and noted that access to trial information varied across providers. In response to these insights, the sponsor developed materials to drive physician awareness (e.g., principal investigator-to-doctor letter and email templates, referral cards with infographic styling, and a study fact sheet) and direct-to-patient multichannel approaches to improve self-driven trial awareness (e.g., study brochure, poster, and social media assets).

When considering eligibility for this study, participants were concerned about concomitant medications and previous drug exposures that may affect their ability to participate. Accordingly, the sponsor amended the clinical trial protocol to expand the allowable previous drug exposures and thus expand the eligible patient population. Participants also had concerns about travel to sites (i.e., distance, time, and expenses), which were alleviated by the sponsor’s offering of travel concierge services and reimbursement of incurred out-of-pocket expenses. To assuage concerns regarding myasthenia gravis crisis management and response time, the site staff reviewed the protocols for management of crises with participants, including the availability of rescue therapies such as plasmapheresis and plasma exchange, and provided direct contact information of site staff should crises occur during the trial.

Participants discussing NCT05556096 also expressed concerns regarding the frequency and duration of study visits, as study activities can be taxing for patients already experiencing weakness. In response, the sponsor added “rests” in between procedures during study visits. Participants expressed a desire for proactive and streamlined communication and were open to technological solutions (e.g., text reminders, apps, websites, and telehealth); they also preferred to complete questionnaires at home prior to study visits. Additionally, despite the sense of independence afforded by self-administration of study drug, participants discussing NCT05556096 had questions on self-administration. To mitigate these concerns, the sponsor developed an instructional video for study drug self-administration and allowed teleconference visits with study sites. Participants wanted to be informed of trial results, study drug efficacy, and whether they received study drug or placebo. These insights required additional discussion to ensure appropriate governance on information sharing; therefore, implementation was postponed.

#### Phase 3 study in pediatric patients with generalized myasthenia gravis (NCT06607627) [1]

Participants reported receiving information on clinical trials from clinic waiting rooms, social media, television commercials, and pediatricians; in response, the sponsor sought to increase trial awareness and foster education in the pediatric clinic setting. Participants, namely parents, asked about the trial requirements surrounding the specification of allowed concomitant therapy, the vaccination requirement, and off-label use of medication in children. Participants shared that the proposed limitation on concomitant medications was not reflective of the real-world patient population. The sponsor deemed that concomitant medications, procedures, and vaccination requirements had to remain as eligibility criteria in the final protocol, though detailed explanations were provided to address concerns and ensure a clear understanding of these criteria and requirements. Participants expressed concern about the number of visits (impact on quality of life) and balancing family obligations; as the trial is in a pediatric population, participants also preferred to avoid school disruption for their children and noted a need for school documentation. In response to these concerns, the sponsor proactively reduced the frequency of clinic visits, increased the number of remote visits during the open-label extension, and removed requirement for morning assessments (to allow flexibility and improve convenience) in the draft clinical trial protocol before finalization. Additionally, the sponsor provided expanded support services including travel, food, hotel, and other accommodations and services for parents and families.

Parents discussing NCT06607627 noted the need for additional support surrounding the device, noting that training and remote access to healthcare providers would be helpful. The discussions also highlighted the importance of play and encouragement around injections in pediatric populations. The sponsor considered how to address this feedback within existing patient support programs.

#### Phase 2 study in adults with active or chronic active AMR after kidney transplantation (NCT06744647) [14]

Participants reported receiving information on clinical trials primarily from social media, internet searches, and other patients, more so than from healthcare providers, and suggested study-specific websites, social media outreach, and paid search campaigns to increase awareness. Participants also noted that visuals regarding AMR or the trial schedule would be helpful for their understanding. In response, the sponsor worked actively with patients to review and select patient-friendly and visually engaging content in recruitment materials (e.g., study brochure describing symptoms, enrollment criteria, the investigational medication, and study timeline) and in-study materials (e.g., a study visit guide with a study summary, reminders, mental health resources, brief descriptions of procedures, a schedule of activities, and a thank you card).

When recalling previous trials, participants discussed transportation and distance to trial sites (e.g., when limited to centers of excellence) as barriers, especially for patients relying on caregivers for transportation. In response to this concern, the sponsor ensured the availability of home healthcare options and travel support for participants who were farther from selected trial sites for ALXN2030-AMR-201. Participants also stressed the importance of regular communication with the trial site and sponsor. To ensure that the visit guide and other in-study materials were comprehensive and easy to follow, the materials were reviewed by the patient advocacy lead and patient representatives to provide feedback on the branding, study names, and overall content. Although participants did not express significant concerns about biopsies every 6 months, they did query about alternative approaches (e.g., molecular biomarkers). To limit the perceived burden of this invasive procedure, the sponsor amended the draft protocol so that biopsies during the open-label extension period were optional and not mandatory. Participants also raised the need for consideration of patients’ mental health while participating in a trial and stressed the importance of showing appreciation and adding a human component to trial participation. The sponsor shared resources (e.g., patient advocacy groups, help lines, and peer programs) with patients and included appreciation cards as part of the in-study materials.

#### Phase 3 study in adults with transthyretin amyloid cardiomyopathy (NCT06183931) [15]

Participants reported receiving information on clinical trials from nurses and other healthcare providers and noted that ClinicalTrials.gov is “too technical.” In response to this insight, the sponsor developed an integrated patient recruitment, advocacy, and healthcare provider awareness plan in patient-friendly language.

Participants wanted clarity about the informed consent form, the rationale for including a placebo arm, how disease progression was going to be assessed, and use of study drug in an extension phase; participants also shared that the exclusion of patients with pacemakers was not reflective of the real-world patient population. The sponsor noted the importance of site staff reviewing clinical trial information with patients to build trust. Frustrations regarding site locations (i.e., sites typically in the same locations) were expressed. The sponsor ensured the availability of travel assistance and concierge services.

Participants requested additional guidance on the goals of the 6-minute walk test (a secondary outcome measure), expressed concerns about the proposed 1:1 (study drug to placebo) design and the contrast magnetic resonance imaging (MRI) procedure (i.e., kidney injury), noted excitement about the study drug mechanism of action (i.e., amyloid depleter), and wanted to avoid multiple blood draws. In response, the sponsor trained trial sites on a standardized approach for the 6-minute walk test (including a script to be read to patients to ensure consistent understanding of the goals), changed the draft protocol to a 2:1 (study drug to placebo) design prior to protocol finalization, clarified the goals of the MRI procedure, and included “amyloid depleter” in the study name.

Participants had questions regarding the route of administration, and this was discussed with the study medical lead. Participants wanted to be informed of study drug efficacy and whether they received study drug or placebo; the sponsor required more discussion to address this need and whether the existing Clinical Trial Transparency processes were sufficient.

## Discussion

Patient centricity is particularly important in the design of clinical trials for rare diseases, as small patient populations complicate the identification, recruitment, and retention of trial participants [16]. We engaged patients, parents of pediatric patients, and patient advocacy group representatives in exchanges about 4 rare disease clinical trials to gather their perspectives early in the development of the clinical trial protocols. In general, these exchanges are a qualitative research method used to obtain feedback on peoples’ behaviors and experiences, and the intimate setting encourages interaction and the exchange of information among participants [17]. This method aligns with the key principles of patient centricity and is particularly apt for discussions on rare diseases, where the population affected by the disease (i.e., patients or caretakers of patients) is small. The inclusion of participants with varying involvements in clinical trial processes and from 6 countries helped ensure that different experiences and global perspectives were given voice. Additionally, these exchanges were intentionally held virtually to accommodate differing time zones, allow flexibility in joining, enable language translation services, and minimize travel obligations for participants. Importantly, 2 of the session participants are authors of this manuscript, highlighting that the expertise and insights they contribute as members of the patient community play a valuable role in informing clinical trial design and should be represented in peer-reviewed publications [7].

Our approach adapted methodology proposed by Cameron et al. for identifying clinical trial protocol design elements that contribute to patient burden (i.e., patient friction) to make patient-centric changes to clinical trial protocols prior to their finalization [18]. Previously, Alexion, AstraZeneca Rare Disease research and development teams explored other quantitative indices for computing protocol complexity and patient burden, but those methods did not incorporate direct qualitative patient input (an expectation with patient engagement [3]) and were unsuitable for application in rare diseases as they relied on numerical data from multiple clinical trials (may not be available or consistent for rare diseases [13]). Thus, the proposed framework incorporates the patients’ voices, qualitatively measures patient impact, and is suitable for rare disease trials while avoiding mathematical calculations that are not feasible in the context of rare diseases and small populations.

Although our approach was used specifically for rare disease clinical trials, we acknowledge that this patient-informed paradigm is applicable for most clinical trials. Additionally, the proposed framework could be expanded to consider challenges that may be specific to individual diseases, such as mobility issues, cognitive issues, or childcare support. For example, a cancer trial that requires regular MRI scanning may allow the use of patients’ recent MRI results at baseline, rather than requiring them to undergo another round of testing thereby decreasing the burden faced by patients in that trial [19].

As of October 17, 2024, this approach of gathering patient and caregiver insights prior to clinical trial protocol finalization was applied during the design of 11 clinical trials, including the 4 described in this paper. Fifteen patient perspective exchange sessions involving participants from 12 countries have been completed. These exchanges led to proactive changes to protocols before they were finalized, including expanding allowable previous drug exposures, telehealth visits, and instructional videos for study drug self-administration, which precluded making amendments. Per Getz et al., this could translate to an estimated cost avoidance of approximately $141,000 and $535,000 per protocol amendment for phase 2 and 3 studies, respectively, and time savings of approximately 3 months per each avoided amendment [20]. Additionally, support services (e.g., travel and other accommodations and mental health resources) continue to be revised in response to the insights received. While these exchanges led to protocol changes, certain patient-derived inputs may not have been fully concordant with regulatory expectations and were therefore not implemented. In these cases, the study sponsors provided education on the limitations to implementing these recommendations including on the regulatory guidance governing drug development.

A key theme from the exchanges was the need for more and improved communication. Participants requested patient-friendly language in materials, additional guidance (regarding eligibility criteria, administration of study drug, and outcome measures), and centralized sources of information to aid organization. As clinical trial protocol amendments are expensive and cause delays [13], actions taken to improve clarity of materials and site staff communications with participants may not only ease participants’ experiences enrolling and participating in these clinical trials but also improve trial efficiency.

This framework serves as an example of how drug developers can collaborate with patient communities and address the calls for action by regulators and patient advocates to engage patients in drug development and throughout the lifecycle of medicines [12]. The last decade has seen an increase in the number of publications on patient engagement, with most patient input being gathered during the design and undertaking of research [12]. While the efforts described here center on patients with rare diseases, other recent initiatives involving patients in the design of clinical trials focus on underrepresented populations (e.g., older adults with Parkinson’s disease and additional comorbidities [19]), vulnerable populations (e.g., patients with cancer undergoing brain radiosurgery [21]), and patients living with pain (e.g., patients with lumbar radiculopathy [22]). There is also interest in integrating digital health technologies into clinical trials to reduce patient burden; areas of focus include digital platforms for data acquisition and real-time analytics, digital biomarkers, outpatient sampling, and smart dosing to improve drug adherence [23]. Adoption of these technologies will require navigation of ethical concerns over patient privacy [24].

Additionally, while our framework centers on collecting patient feedback during protocol development prior to the start of the trial, others have deployed frameworks for soliciting feedback during clinical trials, for example, with the use of sequential patient experience surveys [25]. Similarly, the non-profit organization TransCelerate Biopharma Inc. has developed a toolkit for patient engagement to help standardize sponsors’ approaches [26]. These tools include the Patient Protocol Engagement Toolkit for patient engagement earlier in protocol development and the Study Participant Feedback Questionnaire for patient engagement during clinical studies [27]. Next steps include expanding our initiatives to engage trial participants during rare disease clinical trials in line with the tools outlined above.

Some challenges were identified during planning for the exchanges and subsequent potential protocol changes. These included navigating sponsor governance and compliance policies for patient engagement, ensuring alignment on sharing protocol-level information with participants, and feasibility of amending draft protocols. Future efforts to gain patient insights should, as a standard, seek greater diversity in the participants and engagement with patients who may not already be patient advocates or trained experts. In addition, to demonstrate continued partnership with the participants, and as was done for this work, sponsors should consider including eligible patient participants as authors when publishing their findings.

## Limitations

This initiative has some limitations. The small number of participants included in the patient perspective exchanges and their recruitment through patient advocacy organizations may have restricted the breadth of patient experiences represented. Thus, participants included in the patient perspective exchanges may not fully represent the broader community of patients with these rare diseases. While the sponsor attempted to recruit participants across multiple countries and demographic groups to include a range of perspectives, the sessions were not designed to provide a statistically representative group but rather to obtain qualitative, trial-specific insights to inform trial design. Future studies may examine strategies to recruit more representative samples of patients for such perspective exchanges. Another limitation of this work is the sponsor-led setting, which may have influenced patients and participants’ willingness to share their perspectives openly. To help mitigate this risk, the sessions were designed using patient engagement principles including a collaborative format, a low ratio of sponsor representatives to participants, and confidentiality agreements ensuring feedback was not attributed to individual participants. While the potential for bias cannot be fully excluded, these measures were implemented to encourage candid discussions and facilitate the collection of patient input.

## Conclusions

This work highlights the insights obtained from guided patient perspective exchange sessions across 4 distinct rare disease populations. These discussions led to proactive, patient- and advocate-informed modifications to clinical trial protocols, demonstrating that early patient engagement in rare disease drug development is feasible and can be meaningfully applied to trial design. The success of these exchanges has informed the sponsor’s approach to clinical trial design and patient engagement, consistent with evolving regulatory expectations for incorporating patient perspectives into drug development. Our adapted framework provides a practical example for other organizations seeking to engage patients in the rare disease space and incorporate their perspective into clinical research.

## Supplementary Information

Below is the link to the electronic supplementary material.

Supplementary Material 1

Supplementary Material 2

## Data Availability

Alexion, AstraZeneca Rare Disease, will consider requests for disclosure of clinical study participant-level data provided that participant privacy is assured through methods like data de-identification, pseudonymization, or anonymization (as required by applicable law) and if such disclosure was included in the relevant study informed consent form or similar documentation. Qualified academic investigators may request participant-level clinical data and supporting documents (statistical analysis plan and protocol) pertaining to Alexion-sponsored studies. Further details regarding data availability and instructions for requesting information are available in the Alexion Clinical Trials Disclosure and Transparency Policy at https://www.alexionclinicaltrialtransparency.com/data-requests.

## Declarations

### Generative AI

The first draft of the plain English summary was generated using a generative AI model that distills scientific publications into easy-to-understand summaries. The first draft was reviewed, revised, and approved by the authors. As part of AstraZeneca's commitment to innovation and ethical AI use, this summary was generated within the secure confines of AstraZeneca's infrastructure, ensuring both data privacy and adherence to copyright laws. The authors are accountable for the accuracy and integrity of the final draft of the plain language summary.

### Author's Information

S.B. and P.P are patient participants.

## Abbreviations

AMR: Antibody-mediated rejection
MRI: Magnetic resonance imaging
