# Supplementary material for: Patient engagement in protocol development for rare disease clinical trials: a framework
Source: Res Involv Engagem. 2026 Oct 9;12:167. doi: 10.1186/s40900-026-00985-3 (PMC13651072; doi:10.1186/s40900-026-00985-3)
Supplement: Supplementary file 2 [file 40900_2026_985_MOESM2_ESM.docx]

**Supplementary Materials**

**Patient-engagement in protocol development for rare disease clinical trials: a framework**

Janella Beltran, Sarah Bolton, Pesh Patel, Rohita Sharma

**Table of Contents**

| **Section** | **Title** | **Page** |
| --- | --- | --- |
| Supplemental Table S1 | Clinical trial concept sheets considered during adaptation of the patient friction coefficient | 2 |
| Supplemental Table S2 | Representative semistructured guide for the patient perspective exchange sessions | 7 |
| References | References | 8 |

**Supplemental Table S1.** Clinical trial concept sheets considered during adaptation of the patient friction coefficient

|  | **NCT05556096** [1] | **NCT06607627** [2] | **NCT06744647** [3] | **NCT06183931** [4] |
| --- | --- | --- | --- | --- |
| **Phase** | 3 | 3 | 2 | 3 |
| **Study drug; mechanism** | ALXN1720 (gefurulimab); bispecific antibody that inhibits complement component 5 and binds human serum albumin [5] | Gefurulimab; bispecific antibody that inhibits complement component 5 and binds human serum albumin [5] | ALXN2030; siRNA targeting complement component 3 [6] | ALXN2220; amyloid depleter |
| **Patient population** | Adults with generalized myasthenia gravis with autoantibodies against AChR | Pediatric participants with AChR-Ab+ generalized myasthenia gravis | Adults with AMR after kidney transplantation | Adult participants with transthyretin amyloid cardiomyopathy |
| **Brief disease description** | Myasthenia gravis is an antibody-mediated autoimmune disorder affecting neuromuscular junctions; pediatric and adult patients with a generalized phenotype experience isolated or widespread muscle weakness [7, 8] | | AMR is the most common cause of late graft rejection in kidney transplant recipients, and lack of an FDA-approved treatment contributes to significant heterogeneity in the treatment of this complication [9] | Transthyretin amyloid cardiomyopathy is a systemic amyloidosis (ie, disorder of misfolded precursor protein) and cause of heart failure[10] |
| **Enrollment** | 261 (actual) | 12 (estimated) | 45 (estimated) | 1159 (actual) |
| **Study start**  **(actual)** | November 21, 2022 | November 13, 2024 | March 7, 2025 | January 11, 2024 |
| **Primary completion** | May 27, 2025 (actual) | April 16, 2027 (estimated) | October 11, 2027 (estimated) | June 7, 2027 (estimated) |
| **Study completion (estimated)** | August 31, 2027 | July 18, 2029 | November 7, 2028 | October 5, 2027 |
| **Eligibility criteria** | - Ages ≥18 years - All sexes | - Ages 6–17 years - All sexes | - Ages 18–75 years - All sexes | - Ages 18–90 years - All sexes |
| **Inclusion** | - Diagnosis of myasthenia gravis with generalized muscle weakness meeting the clinical criteria defined by Myasthenia Gravis Foundation of America Class II, III, or IV [11] - Positive serological test for antibodies against AChR | - US-specific:   - Participant must be 12 to <18 years of age at time of providing informed consent   - All participants must be vaccinated against meningococcal infection from serogroups A, B, C, W, and Y within 3 years at least 2 weeks prior to the first dose of study intervention administrations - Rest of world-specific:   - Participants must be 6 to <18 years of age at the time of providing informed consent   - All participants must be vaccinated against meningococcal infection from serogroups A, C, W, and Y (and B where available) within 3 years prior to study intervention on day 1. If vaccination occurs <2 weeks prior to day 1, the participants will receive prophylactic antibiotics for at least 2 weeks after initial meningococcal vaccination for serogroups A, C, W, and Y (and B, where available) - Global:   - Diagnosis of myasthenia gravis with generalized muscle weakness meeting the Myasthenia Gravis Foundation of America Class II, III, or IV [11]   - Positive serological test for autoantibodies against AChR | - Kidney transplant received ≥6 months - Active or chronic active AMR, according to Banff 2022 classification, based on screening kidney biopsy [12] - Either positive C4d on screening kidney biopsy based on the Central Pathology Laboratory report and/or positive HLA Class I and/or II antigen-specific DSA as determined by the local laboratory’s definition of positivity using single-antigen bead based assays - MVI score ≥2 (g ≥1 and ptc ≥1) - eGFR ≥30 mL/min/1.73 m^2^ - Must be vaccinated against meningococcal infection from serogroups A, C, W, Y (and B where available) at least 14 days prior to but no more than 3 years prior to day 1 - Must be vaccinated for *S pneumoniae* prior to randomization - Must be vaccinated for *H influenzae* type B (where available) prior to randomization - Body weight ≥50 kg at screening | - Diagnosis of transthyretin amyloid cardiomyopathy with either wild-type or variant transthyretin genotype - End-diastolic interventricular septal wall thickness ≥12 mm on echocardiography measured at screening - NT-proBNP >2000 pg/mL at screening - Treatment with a loop diuretic for ≥30 days prior to screening - History of heart failure New York Heart Association Class II–IV at screening [13] - Life expectancy of ≥6 months per the investigator's judgment - Males and females of childbearing ability must use contraception |
| **Exclusion** | - History of thymectomy or any other thymic surgery within 12 months prior to screening - Untreated thymic malignancy, carcinoma, or thymoma - History of Neisseria meningitidis infection - Pregnancy, breastfeeding, or intention to conceive during the course of the study | - History of thymectomy or any other thymic surgery within 12 months prior to screening - Untreated thymic malignancy, carcinoma, or thymoma - History of Neisseria meningitidis infection - Pregnancy, breastfeeding, or intention to conceive during the course of the study | - Biopsy-based diagnosis of any of the following at screening:   - TCMR, according to the Banff grade ≥1   - Polyoma virus nephropathy   - Severe thrombotic microangiopathy   - Glomerulonephritis - ABO blood type-incompatible transplant - uACR >2200 mg/g - Multiorgan transplant recipient (except for previous multiple kidney transplants) or cell transplant (islet, bone marrow, stem cell) recipient - Planned or recent treatments, <3 months prior to the screening visit, for Acute Rejection, AMR (including plasmapheresis, plasma exchange, IVIg, B-cell–depleting therapy, IL inhibitors, proteasome inhibitors, high-dose corticosteroids [except for tapering]), TCMR (including T-cell–depleting therapy), excluding the SoC treatment, which will be allowed and should be stable during the entire treatment | - Known leptomeningeal amyloidosis - Known light chain (AL) or secondary amyloidosis (AA) or any other form of systemic amyloidosis - Acute coronary syndrome, unstable angina, stroke, transient ischemic attack, coronary revascularization, cardiac device implantation, cardiac valve repair, or major surgery within 3 months of screening - Uncontrolled clinically significant cardiac arrhythmia, per investigator's assessment - LVEF <30% on echocardiography - Renal failure requiring dialysis or an eGFR <20 mL/min/1.73 m^2^ at screening - Polyneuropathy with PND score IV |
| **Primary outcome measure(s)** | - Change from baseline in Myasthenia Gravis Activities of Daily Living total score at week 26 | - Maximum observed serum concentration (Cmax) of gefurulimab (day 1 predose through week 18 predose) - Serum free complement component 5 concentration (day 1 predose through week 18 predose) | - Biopsy-proven histologic resolution at week 52 | - Total occurrence of all-cause mortality and cardiovascular clinical events during the blinded treatment period (baseline to end of study [up to month 48]) |
| **Locations** |  |  |  |  |
| **Countries** | Argentina, Austria, Brazil, Canada, China, Denmark, France, Germany, Israel, Italy, Japan, Netherlands, Poland, Portugal, Serbia, South Korea, Spain, Switzerland, Taiwan, Turkey, United Kingdom, US | Brazil, Poland, Taiwan, US | Brazil, Canada, China, South Korea, Spain, Taiwan, United Kingdom, US | Argentina, Australia, Austria, Belgium, Brazil, Canada, China, Czechia, Denmark, France, Germany, Greece, Ireland, Israel, Italy, Japan, Netherlands, Norway, Poland, South Korea, Spain, Sweden, Switzerland, Taiwan, Turkey, United Kingdom, US |
| **Total number** | 132 | 12 | 55 | 212 |

ClinicalTrials.gov entries were accessed on December 11, 2025.

AA, serum amyloid A; AChR-Ab+, acetylcholine receptor antibody positive; AL, amyloid light chain; ALT, alanine aminotransferase; AMR, antibody-mediated rejection; AST, aspartate aminotransferase; eGFR, estimated glomerular filtration rate; FDA, US Food and Drug Administration; g, glomerulitis Banff Lesion Score; HIV, human immunodeficiency virus; HLA-DSA, human leukocyte antigen donor-specific antibodies; IL, interleukin; IVIg, intravenous immunoglobulin; LVEF, left ventricular ejection fraction; MVI, microvascular inflammation; NT-proBNP, N-terminal pro–B-type natriuretic peptide; PND, polyneuropathy disability; ptc, peritubular capillaritis Banff Lesion Score; siRNA, small interfering RNA; SoC, standard of care; TCMR, T-cell–mediated rejection; uACR, urine albumin-to-creatinine ratio; ULN, upper limit of normal; US, United States.

**Supplemental Table S2.** Representative semistructured guide for the patient perspective exchange sessions

**Discussion topic 1: Trial awareness**

- What are the primary ways you learn about clinical trials?
- What can sponsors do to make it easier to learn about clinical trials?

**Discussion topic 2: Trial enrollment**

- What factors do you consider when making a decision to participate in a clinical trial?
- What resources or information would be helpful as you consider participating in a clinical trial?
- What can a trial sponsor or CRO do differently to make it easier for patients?

**Discussion topic 3: Trial participation**

- What is your experience with the logistics of a trial (eg, parking, travel, and housing)?
- What is your experience with trial activities (eg, tests, number of and length of visits, home-based questionnaires, schedule of events, and potential self-administration of study drug)?
- What is your social experience of participating in a trial (eg, interactions with trial site staff)?
- Do you have additional concerns?
- How can these challenges be addressed?
- What can a trial sponsor or CRO do differently to make trial participation easier for patients?

**Discussion topic 4: Trial follow-up**

- What is your post-trial experience?
- How can your post-trial experience be improved?

This representative and simplified guide was developed by Alexion, AstraZeneca, Rare Disease.

CRO, clinical research organization.

**References**

1.	Alexion Pharmaceuticals, Inc. Safety and efficacy of ALXN1720 in adults with generalized myasthenia gravis, <https://clinicaltrials.gov/study/NCT05556096> (2022, last revised on 12 June 2025, accessed on 7 August 2025).

2.	Alexion Pharmaceuticals, Inc. PK, PD, safety, and efficacy study of gefurulimab in pediatric patients with AChR+ generalized myasthenia gravis, <https://clinicaltrials.gov/study/NCT06607627> (2024, last revised on 10 July 2025, accessed 7 August 2025).

3.	Alexion Pharmaceuticals, Inc. Phase 2 study of ALXN2030 in patients with antibody-mediated rejection after kidney transplantation (CONCORD), [https://clinicaltrials.gov/study/NCT06744647#study-record-dates](https://clinicaltrials.gov/study/NCT06744647) (2024, last revised on 22 June 2025, accessed 7 August 2025).

4.	Alexion Pharmaceuticals, Inc. Study of ALXN2220 versus placebo in adults with ATTR-CM (DepleTTR-CM), <https://clinicaltrials.gov/study/NCT06183931?intr=ALXN2220&rank=1> (2024, last revised on 1 July 2025, accessed 7 August 2025).

5.	Jindal S, Pedersen DV, Gera N, Chandler J, Patel R, Neill A, et al. Characterization of the bispecific VHH antibody gefurulimab (ALXN1720) targeting complement component 5, and designed for low volume subcutaneous administration. Mol Immunol. 2024;165:29-41.

6.	AstraZeneca. Our pipeline, <https://www.astrazeneca.com/our-therapy-areas/pipeline.html> (Accessed 5 May 2025).

7.	Kaminski HJ, Sikorski P, Coronel SI, Kusner LL. Myasthenia gravis: the future is here. J Clin Invest. 2024;134(12):e179742.

8.	O'Connell K, Ramdas S, Palace J. Management of juvenile myasthenia gravis. Front Neurol. 2020;11:743.

9.	Rodriguez-Ramirez S, Al Jurdi A, Konvalinka A, Riella LV. Antibody-mediated rejection: prevention, monitoring and treatment dilemmas. Curr Opin Organ Transplant. 2022;27(5):405-14.

10.	Ruberg FL, Grogan M, Hanna M, Kelly JW, Maurer MS. Transthyretin myloid cardiomyopathy: JACC state-of-the-art review. J Am Coll Cardiol. 2019;73(22):2872-91.

11.	Task Force of the Medical Scientific Advisory Board of the Myasthenia Gravis Foundation of America, Jaretzki A, 3rd, Barohn RJ, Ernstoff RM, Kaminski HJ, Keesey JC, et al. Myasthenia gravis: recommendations for clinical research standards. Neurology. 2000;55(1):16-23.

12.	Naesens M, Roufosse C, Haas M, Lefaucheur C, Mannon RB, Adam BA, et al. The Banff 2022 kidney meeting report: reappraisal of microvascular inflammation and the role of biopsy-based transcript diagnostics. Am J Transplant. 2024;24(3):338-49.

13.	American Heart Association. Classification of functional capacity and objective assessment, <https://professional.heart.org/en/guidelines-and-statements/classification> (1994, accessed 27 January 2025).
